# Laparoscopic transhiatal suture closure for spontaneous esophageal rupture: a case report

**DOI:** 10.1186/s40792-019-0711-9

**Published:** 2019-10-22

**Authors:** Shunsuke Hayakawa, Akira Mitsui, Yuko Kato, Shota Morimoto, Kaori Watanabe, Tomonari Shamoto, Takehiro Wakasugi, Yoshiyuki Kuwabara

**Affiliations:** Department of General surgery, Nagoya City West Medical Center, 1-1-1 Hirate-cho, Kita-Ku, Nagoya, 462-8508 Japan

**Keywords:** Spontaneous esophageal rupture, Boerhaave’s syndrome, Esophagus, Laparoscopy, Transhiatal approach, Suture closure, Emergency surgery, Conservative treatment, Thoracoscopic surgery, Thoracotomy

## Abstract

**Background:**

Spontaneous esophageal rupture is a rare but serious disease with high mortality. Conservative treatment and endoscopic therapy have been reported, but surgical treatment is still a basic modality of therapy. In addition to thoracotomy, recent studies have reported treatment with thoracoscopic surgery and laparoscopic transhiatal repair. In this study, we report a patient who underwent laparoscopic transhiatal suture closure for spontaneous esophageal rupture with favorable postoperative course. We also discuss indication for laparoscopic surgery for spontaneous esophageal rupture.

**Case presentation:**

A 70-year-old man visited our hospital with chief complaints of epigastric pain and vomitus niger. He was diagnosed with spontaneous esophageal rupture in the left wall of the lower esophagus by computed tomography and upper gastrointestinal (GI) series. At 11 h after the onset of symptoms, we performed laparoscopic transhiatal suture closure and lavage drainage. We performed transhiatal esophageal replacement using the 5-hole approach. We observed a perforation of 2 cm in diameter at the site of the rostral portion approximately 4 cm from the esophageal hiatus. All layers were closed with three stitches using 3–0 absorbable sutures. No perforation was observed in the thoracic cavity. The total operative time was 178 min, and total bleeding was 2 ml. He had no postoperative complications and was discharged on day 15 after the procedure. He received continuous proton pump inhibitor therapy as an outpatient. Healing cicatrization was found at the site of rupture by esophagogastroscopy. The patient was advised to improve his lifestyle and has shown no signs of recurrence over 2 years from the date of surgery.

**Conclusions:**

Simple closure of all the layers using laparoscopic transhiatal simple closure was useful in the treatment of esophageal rupture as a less invasive approach for patients who meet the following conditions: stable general condition, intrathoracic perforation, and the perforation site is identified as the lower esophagus by pre-operative examination.

## Background

Spontaneous esophageal rupture is a rare but serious disease with mortality of 20–40%. Surgical treatment is still a basic modality of therapy for this disease, but conservative treatment and endoscopic therapy can be also chosen. The treatment strategy should be decided carefully according to individual disease severity and the site of perforation. In addition to the conventionally performed thoracotomy, thoracoscopic surgery has also been reported [1–3]. For patients with a perforation in the lower esophagus, laparoscopic transhiatal simple closure is a viable alternative therapy. In this study, we report a patient who underwent suture closure of laparoscopic transhiatal simple closure with favorable postoperative course. We also discuss indication for laparoscopic surgery for spontaneous esophageal rupture.

## Case presentation

A 70-year-old man visited our emergency unit with chief complaints of epigastric pain and vomitus niger 2 h after the onset of symptoms. After consultation in our hospital, the patient had black vomit. His blood pressure was 168/109 mmHg, pulse was 73 bpm, and body temperature 36.7 °C. For abdominal findings, he had rebound tenderness, although tenderness was present in the epigastrium. With regard to significant medical history, he had Mallory–Weiss syndrome, high blood pressure, and hyperlipidemia detected 14 years ago, as well as removal of *Helicobacter pylori* 2 years prior. For relevant lifestyle history, the patient claims to drink five cups of coffee, consumes 360 cc of alcohol each day, and is currently a non-smoker (quit smoking 10 years ago). He is currently medicated with oral antihypertensive agents. Results of his blood tests revealed white blood cell count of 13,560/ml and C-reactive protein of 0.3 mg/dl.

**Fig. 1:**
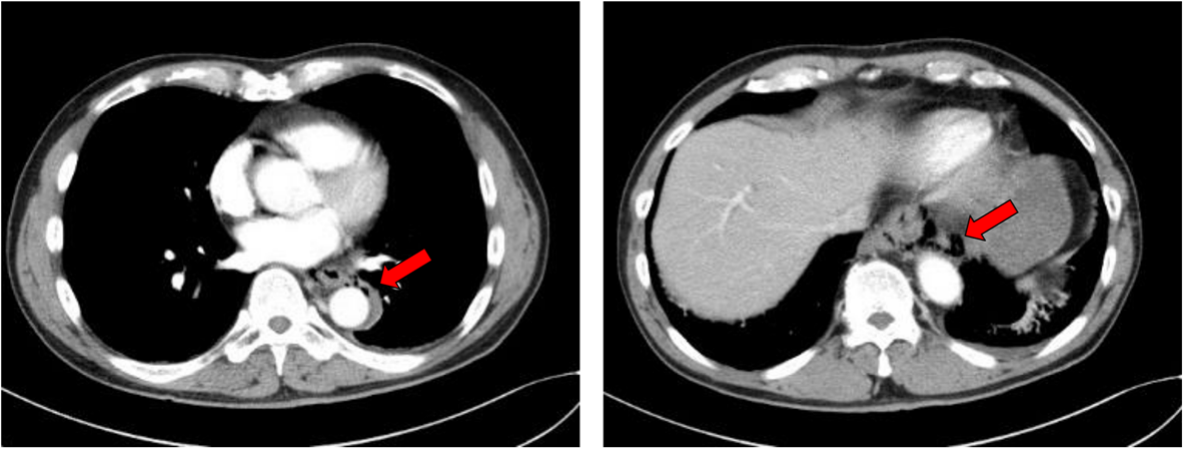
Computed tomography (CT) scan. CT revealed pneumomediastinum extending from the upper surface of the diaphragm to the aortic arch

**Fig. 2:**
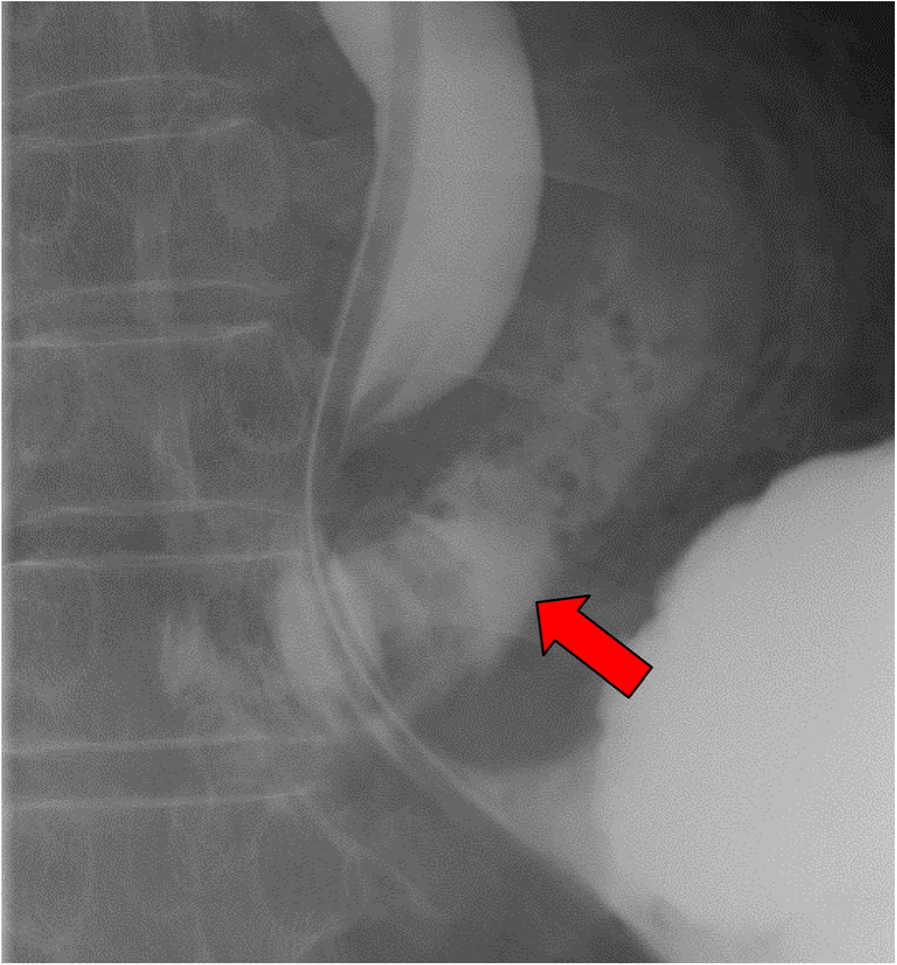
Contrast study. Red arrow shows the extravasation of contrast to the mediastinum

Contrast computed tomography (CT) revealed empyema and fluid retention in the mediastinum (Fig. 1). There were no abnormal findings such as fluid retention and pneumothorax in the thoracic cavity. Upper GI series (with Urografin) detected leakage of contrast media into the mediastinum from the lower intrathoracic esophagus on the left side (Fig. 2). We repeated plain CT scans following the upper GI series and confirmed leakage of contrast media into the mediastinum from the site just above the cardia to the tracheal bifurcation. Based on the above findings, the patient was diagnosed with spontaneous esophageal rupture and we decided to perform emergency surgery. Because the location of the perforation was suspected to be the left side of the lower esophagus, the patient’s vital sign was stable, and the area of perforation was localized to the mediastinum, laparoscopic transhiatal simple closure was chosen. We performed the procedure 11 h after the onset of symptoms.

**Fig. 3:**
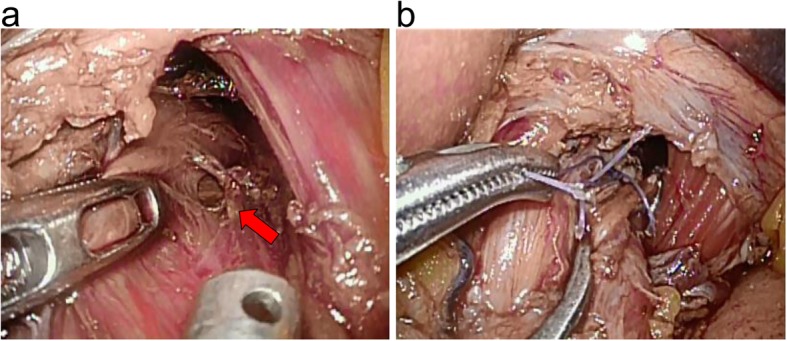
Intraoperative view. **a** Red arrow shows the site of perforation. **b** The rupture site was successfully closed with simple sutures

The ports were inserted using the 5-hole approach. No abnormal findings were found in the abdominal cavity. It was found that contamination was mainly on the left side, and we initially searched for perforation placement from the left side of the esophagus. We removed a volume of tissue equivalent to three quarters of the circumference of the esophagus from the dorsal esophagus to the right dorsal esophagus. A perforation of 2 cm in diameter was observed at the site of the rostral portion at approximately 4 cm from the esophageal hiatus (Fig. 3a). No intrathoracic perforation was observed. Endoscopy of the upper gastrointestinal tract was performed during surgery, and from the esophageal lumen, it was confirmed that this was the site of perforation. The perforation was closed with three stitches using 3–0 absorbable sutures (Fig. 3b). Before concluding surgery, the mediastinum was irrigated with saline and two drains were inserted into the subdiaphragm around hiatal space transperitoneally. The total operative time was 178 min, and the total volume of bleeding was 2 ml.

**Fig. 4:**
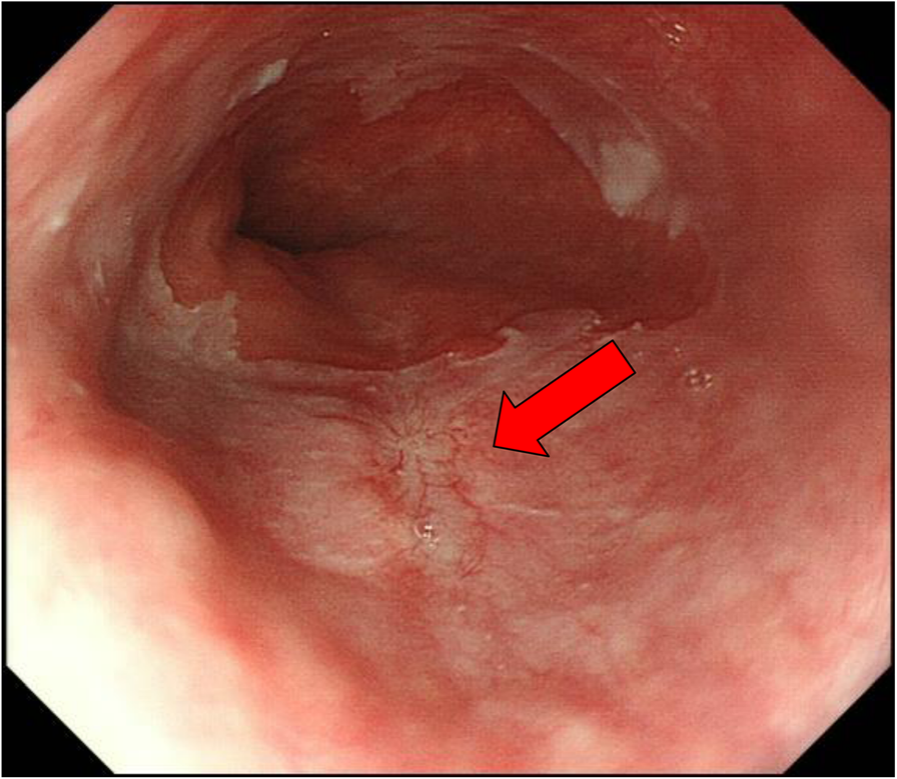
Endoscopic view (3 months after operation). Red arrow shows scar formation

After surgery, the two drains were used for intermittent suction, and we continued treatment with proton pump inhibitor (30 mg, 2 times a day) and antibiotics (MEPM 1 g, 3 times a day). The patient started ambulation on the first postoperative day. His white cell count was normalized on postoperative day 4. The gastric tube was removed with fluoroscopic guidance. Antibiotics were discontinued on postoperative day 6. Oral intake of food was initiated on postoperative day 7, and after shifting meals to a solid diet, recovery was uneventful. The patient was discharged on postoperative day 15 and continued proton pump inhibitor therapy as an outpatient. Healing cicatrization was found at the site of rupture by endoscopy of the upper gastrointestinal tract (Fig. 4). The patient was advised to improve his lifestyle. He showed no signs of recurrence two or more years after surgery.

## Discussion

Boerhaave’s syndrome is an esophagus rupture which occurs idiopathically and was first reported by Herman Boerhaave in 1724 [4]. It is a serious disease with a mortality rate of 20–40%, and in many cases, a perforation is located on the left side of the lower third of the esophagus [5]. The treatment method varies depending on disease severity; conservative treatment, gastrointestinal endoscopic therapy, and surgical therapy are available. Surgical therapy is generally classified into transthoracic or transhiatal procedures.

Recent reports have demonstrated the usefulness of conservative treatment for esophagus rupture [6, 7]. Its indication has been reported by Cameron et al. and Altorjay et al. with the recommendation that close attention should be paid with regard to sepsis under the following conditions: the lesion is localized, no tumor tissue is found, no perforation is found in the abdominal cavity, and no obstructive esophageal disease is found [8, 9]. However, all of these cases were based on a retrospective study performed on a small sample. In the clinical setting, physicians must make a decision based on individual symptoms. Furthermore, some studies on endoscopic treatment have also been reported. Although they examined a small sample, patients underwent treatment by clipping the perforation under endoscope guidance, and recovery of these patients followed a more benign course [10, 11].

It has also been reported that an esophagus stent provided good results for iatrogenic esophageal perforation and failure of the sutures after simple closure. However, some reports showed more fatal outcome with stentinding than surgical treatment [12, 13]. Spontaneous esophageal rupture has also been reported to have increased mortality if proper intervention is not performed within 24 h of onset [14]. There is a risk of missing the optimal timing for surgery if the conservative treatment is not effective. Non-surgical treatment including endoscopic therapy should be considered carefully in the context of the individual patient symptoms because these methods may miss an opportunity which is best suited for surgery.

As previously described, the usefulness of conservative treatment has also been examined, but surgical treatment is still a basic modality of therapy for this disease [10]. The purpose of surgical treatment includes a lavage drainage and suture closure of the perforation, and in some cases, construction of gastric fistula and intestinal fistula depending on the clinical condition of the patient. To decide on an operative method, it is extremely important to identify the location of the perforation. Conventionally, thoracotomy is mainly performed, and recently, the number of reports on thoracoscopic surgery has increased [1–3]. Cho et al. showed that results of thoracoscopic surgery were not inferior to thoracotomy. Nakano et al. reported no difference in surgery results of laparotomy between patients who underwent primary suture with omentopexy via a laparotomy followed by left thoracoscopic mediastinal drainage and patients who underwent left thoracoscopic primary suture and mediastinal drainage [3]. Thoracoscopic surgery is becoming common and effectual therapy for Boerhaave’s syndrome.

However, reports of laparoscopic transhiatal simple closure approach are scarce. Landen et al. used a peritoneoscope in three patients with Boerhaave’s syndrome as a minimally invasive approach [15] with good postoperative course in one patient, one patient dying of sepsis, and one patient developed failure of the sutures. Intrathoracic perforation was suspected in the patient who died and the patient with failure of the sutures by CT scan. Ashrafi et al. used peritoneoscope combined with thoracoscope for patients with intrathoracic perforation, performing a two-layer repair for the perforation. The postoperative course was uneventful, and he was discharged on day 9 after the procedure [16]. Mikami et al. performed a hand-assisted laparoscopy in a patient with perforation localized in the mediastinum, performed simple closure of the perforation, and inserted drains. The patient’s postoperative course was uneventful, and he was discharged on week 3 after the procedure [17]. Kimberley et al. inserted drains into the left pleural cavity in addition to transperitoneal drain insertion, as well as suture closure laparoscopically. The patient was discharged on day 15 after the surgery, and they concluded that the laparoscopic approach should be performed for patients without sepsis [18].

Recently, transperitoneal laparoscopic surgery has been performed frequently in patients with esophagogastric junction cancer, and the opportunity to gain experience in such surgery around the lower esophagus has also increased. It is now thought that surgeons who have learned the techniques of removal or replacement of the esophagus and suture in a narrow space can attempt laparoscopic surgery for Boerhaave’s syndrome. This method can avoid thoracotomy in patients without intrathoracic perforation such that a less invasive treatment can be provided because thoracic cavity drain is not required.

Although the indication for laparoscopic surgery should be considered carefully, it should be considered for patients who meet the following conditions: no sepsis, stable general condition, no intrathoracic perforation is observed, and the location of perforation is identified as the lower esophagus. This procedure should be limited to cases within 24 h of onset. It is believed that this procedure is not positively recommended, considering the risk of complications in cases that do not meet the aforementioned criteria. In our patient, we laparoscopically confirmed no intrathoracic perforation during his surgery.

Laparoscopic transhiatal simple closure is a more reliable procedure than non-surgical treatment because we can observe the site of perforation. And this procedure is less invasive than thoracotomy for Boerhaave’s syndrome compared to conservative treatment after being carefully considered.

## Conclusions

Simple closure of all the layers using laparoscopic transhiatal simple closure is considered as a viable less invasive operative procedure for the treatment of esophageal rupture for patients who meet the following conditions: stable general condition, absence of intrathoracic perforation, and the perforation site is identified as the lower esophagus by pre-operative examination.

## Data Availability

The datasets supporting the conclusions of this article are included within the article.

## Abbreviations

CT: Computed tomography
GI: Gastrointestinal
MEPM: Meropenem
